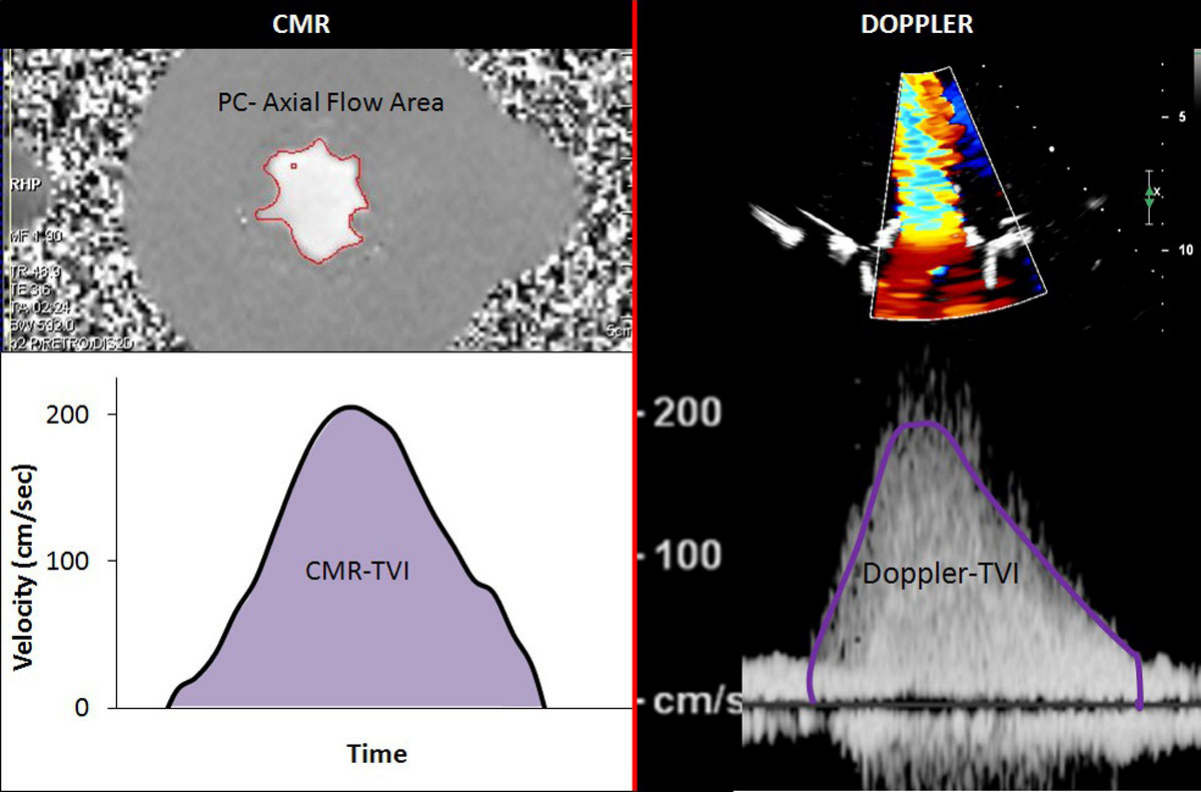# Bioprosthetic mitral valve effective orifice area by phase-contrast CMR. An in vitro comparison with Doppler echocardiography

**DOI:** 10.1186/1532-429X-16-S1-T3

**Published:** 2014-01-16

**Authors:** Dimitrios Maragiannis, Matthew Jackson, Karen Chin, Kyle Autry, Stephen Igo, Dipan J Shah, Stephen H Little

**Affiliations:** 1 Cardiac MRI, Houston Methodist Hospital, Houston, Texas, USA

## Background

Introduction: Current guidelines for the functional evaluation of bioprosthetic heart valves recommend the effective orifice area (EOA) as the product of the trans-valvular stroke volume divided by Doppler derived diastolic time velocity integral (TVI). Phase contrast CMR may offer an alternative imaging modality to assess bioprosthetic valve EOA when Doppler methods are technically limited or unreliable.

## Methods

Our circulatory loop includes a mock ventricle and a heart valve imaging chamber that has been fabricated using MRI-compatible components. In this study 3 different sized stented porcine mitral valve bioprostheses were evaluated (27 mm, 29 mm, 31 mm) replicating three different hemodynamic conditions with forward stroke volume of 70 ml, 90 ml and 110 ml respectively at a beat rate of 70 bpm. Imaging was performed with a 1.5T Siemens Avanto scanner. CMR imaging parameters consisted of 25 phases, slice thickness 4 mm, spatial resolution of 138 × 256 and temporal resolution of 49 msec. Phase contrast pulse sequences were acquired and peak instantaneous velocities were plotted throughout the diastolic filling period. Stroke volume was measured by MRI-compatible high fidelity flow transducer. The velocity time integral of peak velocity was calculated by tracing the area under the curve and CMR-EOA (cm2) was calculated by dividing the forward flow (cm3) by the MRI-derived TVI (cm). Doppler derived EOA was determined by dividing the stroke volume by the continuous wave Doppler TVI.

## Results

Bioprosthetic mitral valve diastolic flow area was assessed for 3 different sized valves each at 3 flow volume conditions (N = 9). Doppler-EOA and CMR-EOA were measured and compared for each condition. CMR-derived TVI demonstrated a strong and statistically significant correlation with Doppler-derived TVI (r = 0.97, p < 0.001). CMR-EOA also revealed a strong and statistically significant correlation with Doppler derived EOA (r = 0.90, p = 0.001). Mean EOA difference was 0.2 cm2 ± 0.13. The lower temporal resolution of phase contrast CMR velocity determination may have led to the lower TVI values and slightly larger EOA calculation compared to Doppler TVI method

## Conclusions

In this study we report a novel method to determine mitral prosthetic valve effective orifice area using phase contrast CMR values. We demonstrate a strong correlation with the current Doppler standard method to derive EOA. CMR-derived EOA may be an important parameter of prosthetic valve function when Doppler methods are unobtainable or unreliable.

## Funding

AHA beginning grant in aid.

**Figure 1:**